# Supplementary material for: mRNA Turnover Protein 4 Is Vital for Fungal Pathogenicity and Response to Oxidative Stress in Sclerotinia sclerotiorum
Source: Pathogens. 2023 Feb 8;12(2):281. doi: 10.3390/pathogens12020281 (PMC9960052; doi:10.3390/pathogens12020281)
Supplement: Supplementary file 1 [file pathogens-12-00281-s001.zip › pathogens-2201314-supplementary.pdf]

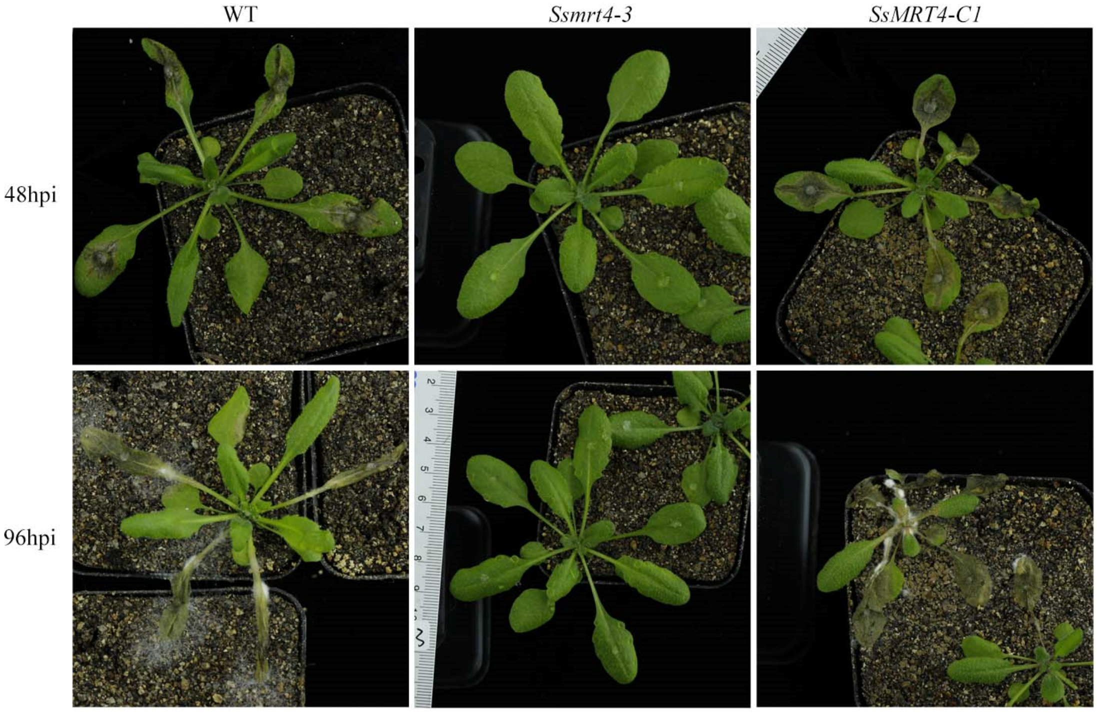

Figure S1. Lesion symptoms of WT, *Ssmrt4-3* and *SsMRT4-C1* on undetached leaves of *A. thaliana* at 48hpi and 96hpi.

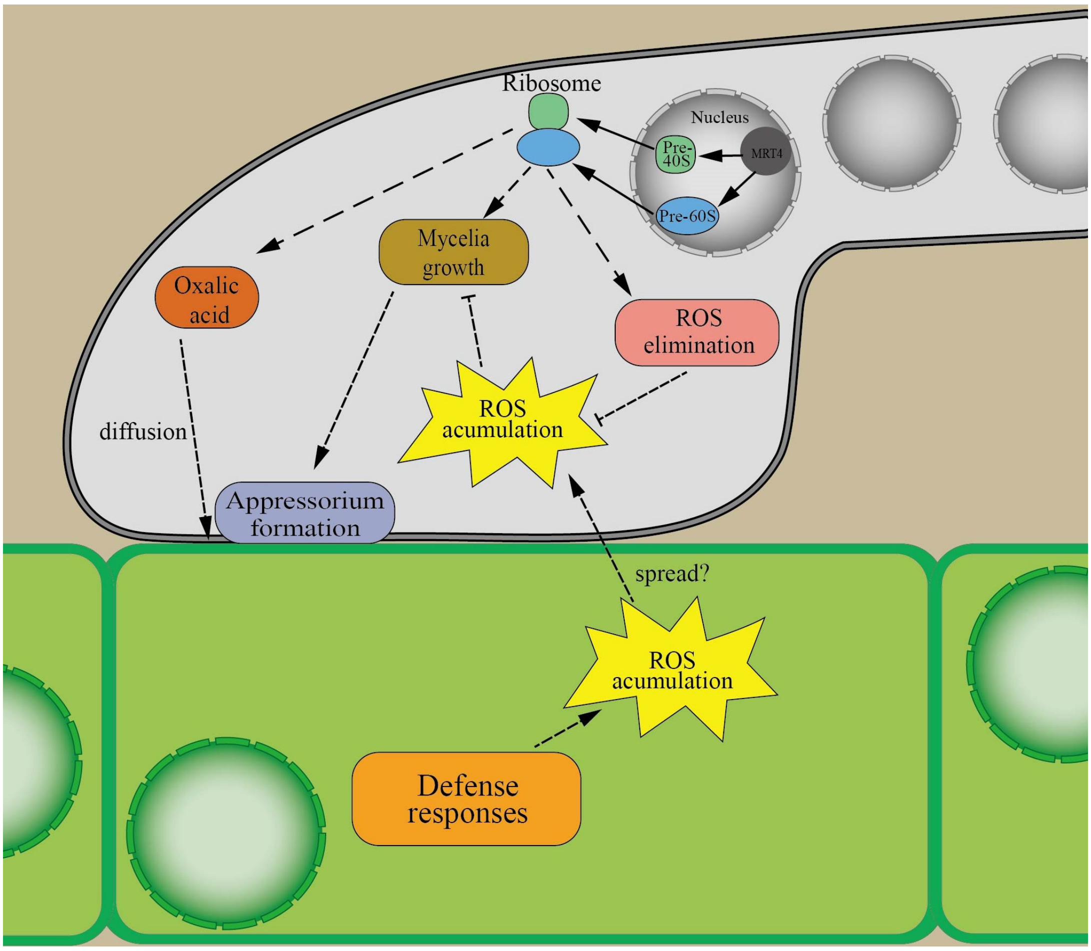

Figure S2. A model that reveals the function of *SsMRT4* in *S. sclerotiorum*. *Ss*MRT4 influences the accumulation of the pre-60S subunit as a ribosomal

transacting factor, thereby affecting the assembly of immature ribosomes in nucleus. The failure to form mature proteins seriously interferes with the formation of appressorium and secretion of oxalic acid, resulting in complete loss of pathogenicity of the mutant. Furthermore, both endogenously produced and exogenously accumulated ROS cannot be eliminated in time, which further affects the growth and development of *S. sclerotiorum*.
